# Treatment of Unstable Thoracolumbar Fractures through Short Segment Pedicle Screw Fixation Techniques Using Pedicle Fixation at the Level of the Fracture: A Finite Element Analysis

**DOI:** 10.1371/journal.pone.0099156

**Published:** 2014-06-10

**Authors:** Changqing Li, Yue Zhou, Hongwei Wang, Jun Liu, Liangbi Xiang

**Affiliations:** 1 Department of Orthopedics, Xinqiao Hospital, the Third Military Medical University, Chongqing, China; 2 Department of Orthopedics, General Hospital of Shenyang Military Area Command of Chinese PLA, Shenyang, Liaoning, China; Toronto Western Hospital, Canada

## Abstract

**Objective:**

To compare the von Mises stresses of the internal fixation devices among different short segment pedicle screw fixation techniques to treat thoracic 12 vertebral fractures, especially the mono-segment pedicle screw fixation and intermediate unilateral pedicle screw fixation techniques.

**Methods:**

Finite element methods were utilised to investigate the biomechanical comparison of the four posterior short segment pedicle screw fixation techniques (S4+2: traditional short-segment 4 pedicle screw fixation [SPSF]; M4+2: mono-segment pedicle screw fixation; I6+2: intermediate bilateral pedicle screw fixation; and I5+2: intermediate unilateral pedicle screw fixation).

**Results:**

The range of motion (ROM) in flexion, axial rotation, and lateral bending was the smallest in the I6+2 fixation model, followed by the I5+2 and S4+2 fixation models, but lateral bending was the largest in the M4+2 fixation model. The maximal stress of the upper pedicle screw is larger than the lower pedicle screw in S4+2 and M4+2. The largest maximal von Mises stress was observed in the upper pedicle screw in the S4+2 and M4+2 fixation models and in the lower pedicle screw in the I6+2 and I5+2 fixation models. The values of the largest maximal von Mises stress of the pedicle screws and rods during all states of motion were 263.1 MPa and 304.5 MPa in the S4+2 fixation model, 291.6 MPa and 340.5 MPa in the M4+2 fixation model, 182.9 MPa and 263.2 MPa in the I6+2 fixation model, and 269.3 MPa and 383.7 MPa in the I5+2 fixation model, respectively. Comparing the stress between different spinal loadings, the maximal von Mises stress of the implants were observed in flexion in all implanted models.

**Conclusion:**

Additional bilateral pedicle screws at the level of the fracture to SPSF may result in a stiffer construct and less von Mises stress for pedicle screws and rods. The largest maximal von Mises stress of the pedicle screws during all states of motion were observed in the mono-segment pedicle screw fixation technique.

## Introduction

Approximately 90% of spinal fractures are found in the thoracolumbar segment, and burst fractures comprise 10%–20% of such injuries [1], [2]. The aims of surgery in these fracture cases include decompression of the neural components, fracture reduction, and providing a stable fixation until arthrodesis is achieved. Short-segment spinal instrumentation has been beneficial in the management of thoracolumbar spinal fractures for better correction of kyphotic deformity, greater initial stability, early painless mobilisation, and indirect decompression of the spinal canal [3], [4]. Despite the advantages of this approach, it is also associated with loss of reduction and instrumentation failure in some cases [3]–[6]. In recent years, a large amount of biomechanical and clinical evidence has suggested that reinforcement with fracture-level screw combinations can help to improve the biomechanical stability and provide better kyphosis correction [7]–[12], but no studies have compared the von Mises stresses of the internal fixation devices among different short segment pedicle screw fixation techniques using pedicle fixation at the level of the fracture, especially the mono-segment pedicle screw fixation and intermediate unilateral pedicle screw fixation techniques [13]–[16]. Through the study of von Mises stresses of the internal fixation devices among different short segment pedicle screw fixation techniques, we can speculate the possibility of screw breakage among different fixation models.

In this study, we investigated the biomechanical comparison of the four posterior short segment pedicle screw fixation techniques (S4+2: traditional short-segment 4 pedicle screw fixation [SPSF]; M4+2: mono-segment pedicle screw fixation; I6+2: intermediate bilateral pedicle screw fixation; I5+2: intermediate unilateral pedicle screw fixation) used in thoracolumbar burst fractures. We developed a finite element model of the intact thoracolumbar junction from T9 to L3 with thoracic 12 vertebral fracture, and various spinal fusion models were developed using the intact model. The range of motion (ROM), von Mises stress, and stress nephogram of the pedicle screws and rods were evaluated.

## Materials and Methods

The software Mimics 10.0 (Materialise, Belgium), Rapidform 2006 (INUS, Korea), and Abaqus 6.9 (Simulia, USA) were used. A computed tomography scanner (GE, USA) was used to collect raw data in DICOM format with a scan slice of 0.625 mm.

### The intact and fracture model

**Figure 1:**
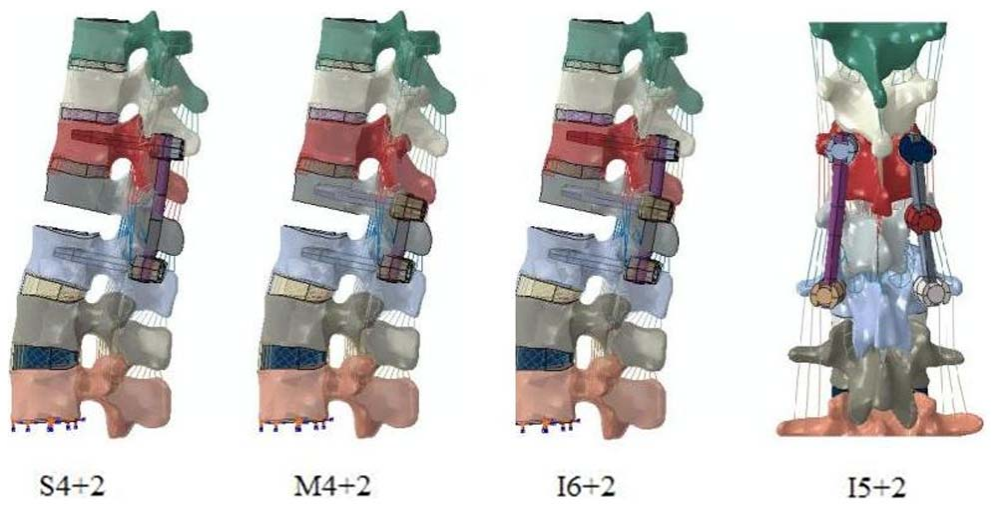
Four finite element models with T9-L3 spine segments. S4+2 means traditional short-segment pedicle screw fixation, M4+2 means mono-segment pedicle screw fixation, I6+2 means intermediate bilateral pedicle screw fixation, and I5+2 means intermediate unilateral pedicle screw fixation.

A finite element model of the T9-L3 spine, which included seven vertebras and six discs, was reconstructed. Geometrical details of T9-L3 spine vertebras were obtained from 64 spiral computed tomography (CT) images of a 40 years old healthy male (65kg and 175cm) without a history of spine injury, osteoporosis and radiographic evidence of degenerative sign. The CT images were scanned and imported into Mimics 10.0 (Materialise, Belgium). The surface model was then exported into Rapidform 2006 (INUS, Korea) to generate and enhance the quality of the solid model. Eventually, it was imported to Abaqus 6.9 (Simulia, USA) for meshing and analysis. Each vertebral body consisted of cortical bone and cancellous bone, and each vertebral disc was composed of nucleus pulposus, annulus fibrosus, and endplates. Posterior elements were built separately from the vertebral bodies. Based on Boolean operation, the lower half of the T12 segment was resected, and the structure of the posterior part was reserved to establish a finite element model of an unstable thoracolumbar fracture (**Fig. 1**).

### Fixation models

Four fixation models were established using the unstable thoracolumbar fracture: the S4+2 model, M4+2 model, I6+2 model, and I5+2 model. In the current study, S4+2 means traditional short-segment pedicle screw fixation, M4+2 means mono-segment pedicle screw fixation, I6+2 means intermediate bilateral pedicle screw fixation, I5+2 means intermediate unilateral pedicle screw fixation. The implants and the vertebral structure were precisely engaged. The diameter of the screw was 6 mm, and the total length of the screw was 45 mm. The fixation models are shown in **Fig. 1**. The element types, material properties, and ligamentary cross-sectional area are shown in **Table 1** [17]–[20].

**Table 1:** Material properties in the present FEM.

Component	Young's modulus (MPa)	Poisson's ratio	Cross section (mm^2^)
Cortical bone and endplate	12000	0.30	
Cancellous bone	100	0.20	
Annulus	4.2	0.40	
Nucleus pulposus	1	0.49	
Anterior longitudinal ligaments	7.8	0.40	63.7
Posterior longitudinal ligaments	10	0.30	20
Supraspinous and interspinous ligaments	10	0.30	70
Ligamentum flavum	15	0.30	40
Intertransverse ligament	10	0.30	1.8
Capsular ligament	7.5	0.30	30
Pedicle screws and rods	110000	0.30	

### Boundary and loading conditions

The inferior endplate of L5 was constrained in all degrees of freedom. A pure moment of 10 Nm combined with a pre-compressive load of 150 N [19], [21]–[23] was applied to the top surface of T9. Flexion, extension, left/right lateral bending, and left/right axial rotation were simulated.

### Rationalities of the models

To validate the rationalities of the models, including model simplification, material properties, boundary conditions, and loads, a moment of 10 Nm and a compressive load of 150 N were applied to the reference point. These loading conditions are adopted from the biomechanical experiments and published finite element analyses [21]–[24]. The range of motion (ROM) among different models was compared (**Table 2**). There is little difference between the models. Therefore, the models in the present study are effective for further analyses.

**Table 2:** Comparison between the current intact model and a previous study.

States of motion	ROM of L1/2 (°)		ROM of L2/3(°)		Mean value of ROM(°)	
	Present study	Yamamoto's study [21]	Present study	Yamamoto's study [21]	Present study	Pflugmacher's study [24]
Flexion	5.9	5.8±0.6	5.5	6.5±0.3	4.6±0.6	5.3±1.0
Extension	4.4	4.3±0.5	5.4	4.3±0.3	4.5±1.1	5.7±1.0
Left axial rotation	3.0	2.6±0.5	2.8	2.2±0.4	3.2±0.8	2.1±0.5
Right axial rotation	3.8	2.0±0.6	2.9	3.0±0.4	3.2±0.6	2.1±0.5
Left lateral bending	6.1	5.2±0.4	5.3	7.0±0.6	4.6±0.7	4.3±0.6
Right lateral bending	6.1	4.7±0.4	6.5	7.0±0.6	4.8±0.5	4.3±0.6

### Assessment indexes

The range of motion (ROM) of T11-L2, von Mises stress, and stress nephogram of the pedicle screws and rods of the four fixation finite element models under six loading (flexion, extension, left/right lateral bending, and left/right axial rotation) conditions were analysed. No statistical analysis was performed in the manuscript because only one subject was modeled.

## Results

### Range of motion (ROM)

**Figure 2:**
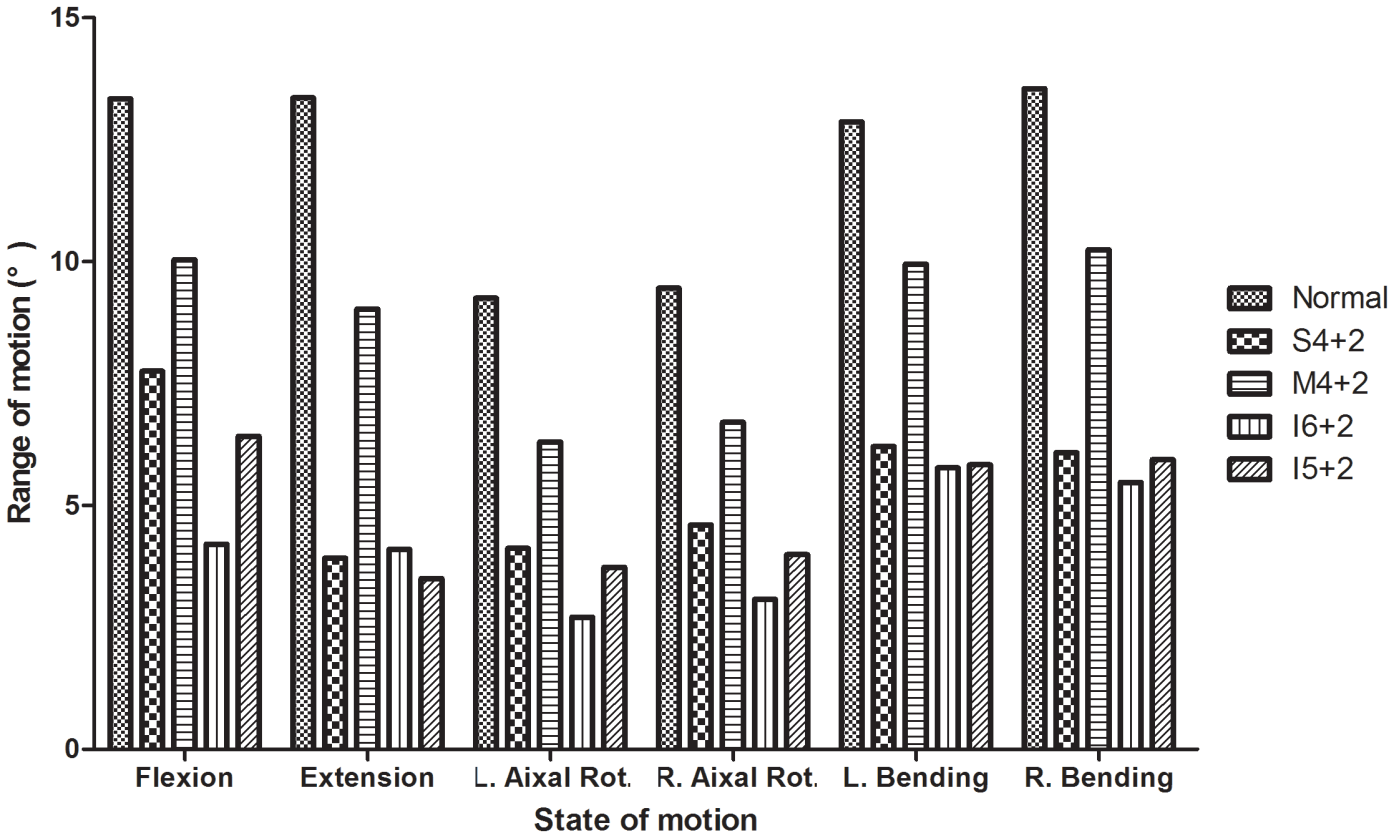
The results of range of motion (ROM). Compared with the intact normal spine model, the four fixation models showed a decreased range of motion (ROM) in all states of motion.

Compared with the intact normal spine model, the four fixation models showed a decreased range of motion (ROM) in all states of motion, as shown in **Fig. 2**. The ROM in flexion, axial rotation, and lateral bending was the smallest in the I6+2 fixation model, followed by the I5+2 and S4+2 fixation models, but the ROM was the largest in the M4+2 fixation model. In the case of I6+2, the ROM was 31.6% in flexion and 30.6% in extension of that of the intact spine. In the case of I5+2, the ROM was 48.1% in flexion and 26.1% in extension of that of the intact spine. In the case of M4+2, the ROM was 75.2% in flexion and 67.2% in extension of that of the intact spine. In axial rotation, the ROM could be ranked in the following order from small to large: I6+2, I5+2, S4+2, and M4+2. Of particular interest, the ROM in I6+2 is similar to that of the other fixation models, except for the M4+2 fixation model.

### von Mises stress of the pedicle screws

**Figure 3:**
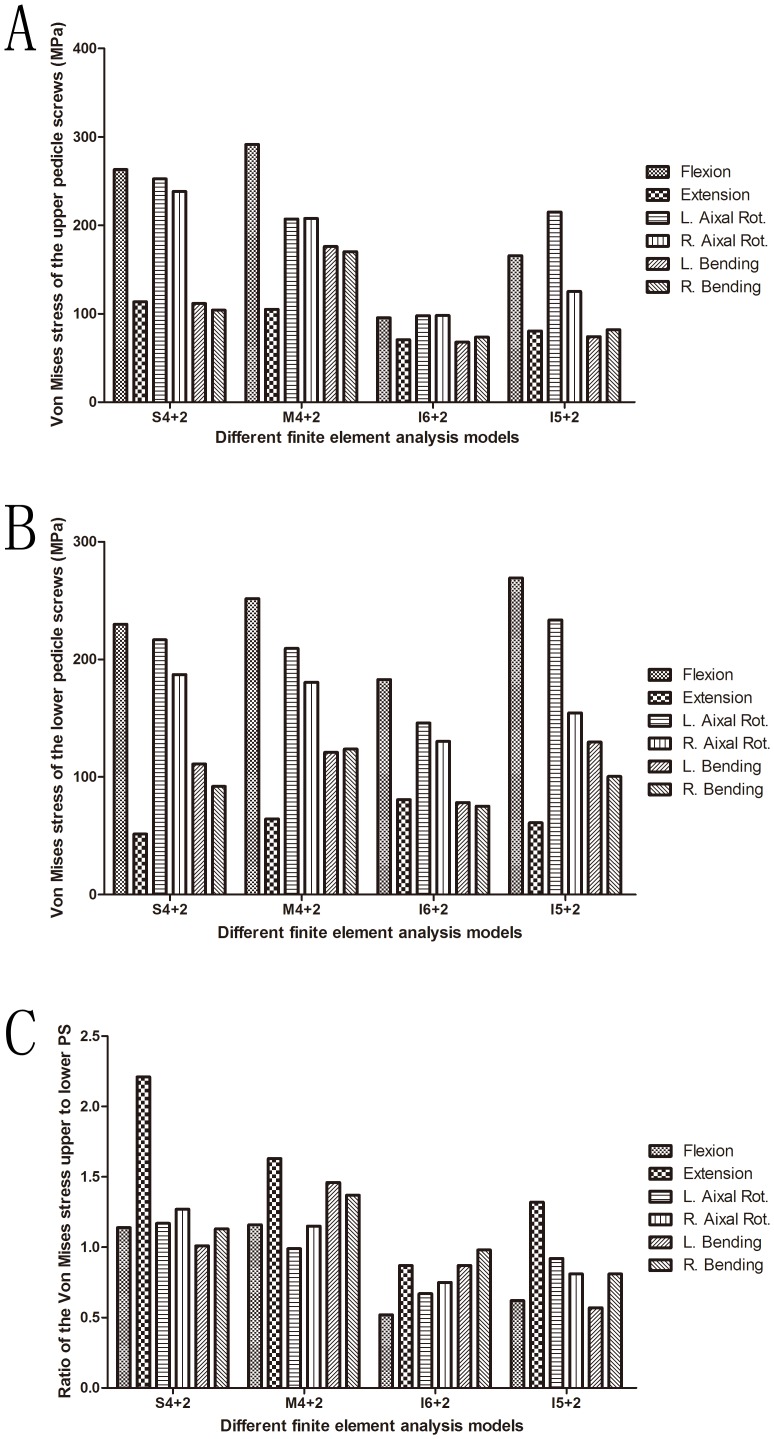
von Mises stress of the pedicle screws. A. von Mises stress of the upper pedicle screws. B. von Mises stress of the lower pedicle screws. C. Ratio of the von Mises stress of the upper pedicle screws to that of the lower pedicle screws.

The largest maximal von Mises stress of pedicle screws all happened in flexion among all of the fixation models during all states of motion (**Fig. 3**). The maximal stress of the upper pedicle screw is larger than that of the lower pedicle screw in S4+2 and M4+2. The largest maximal von Mises stress happened in the upper pedicle screw in the S4+2 and M4+2 fixation models and in the lower pedicle screw in the I6+2 and I5+2 fixation models (**Fig. 3**). The values of the largest maximal von Mises stress of the pedicle screws during all states of motion were 263.1 MPa, 291.6 MPa, 182.9 MPa, and 269.3 MPa in the S4+2, M4+2, I6+2, and I5+2 fixation models, respectively. Comparing the stress between different spinal loadings, the maximal von Mises stress of the implants were observed in flexion in all implanted models. According to the I5+2 fixation model, there was difference in the von Mises stress of the pedicle screw between left and right axial rotation about more than 60 MPa.

### von Mises stress of the rods

**Figure 4:**
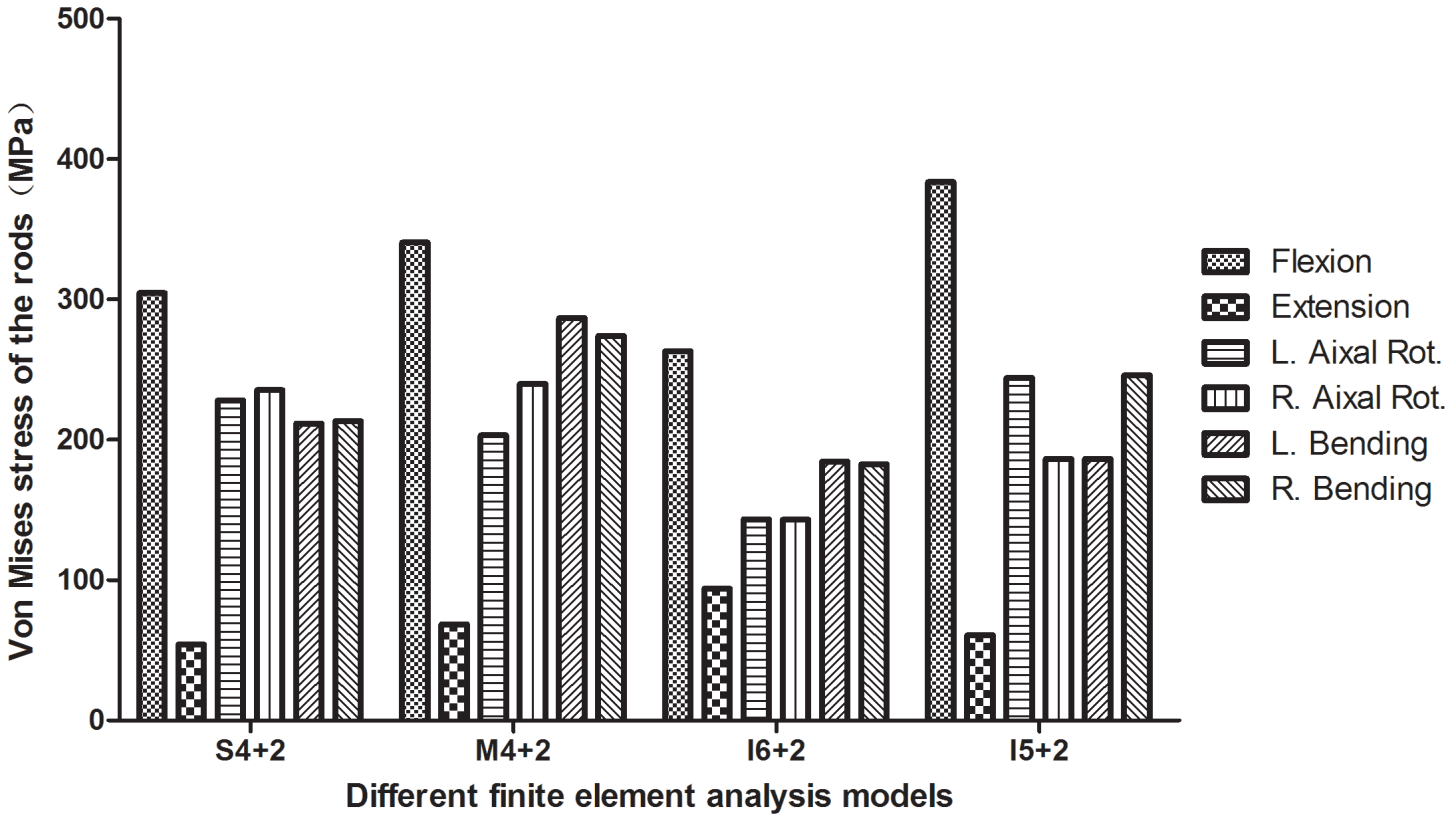
von Mises stress on the rods. The largest maximal von Mises stress on the rods all happened in flexion among all of the fixation models during all states of motion.

The largest maximal von Mises stress of rods all happened in flexion among all of the fixation models during all states of motion (**Fig. 4**). The values of the largest maximal von Mises stress of the rods during all states of motion were 304.5 MPa, 340.5 MPa, 263.2 MPa, and 383.7 MPa in the S4+2, M4+2, I6+2, and I5+2 fixation models, respectively. Comparing the stress between different spinal loadings, the maximal von Mises stress of the implants were observed in flexion in all implanted models.

### Stress nephogram of the pedicle screws and rods

**Figure 5:**
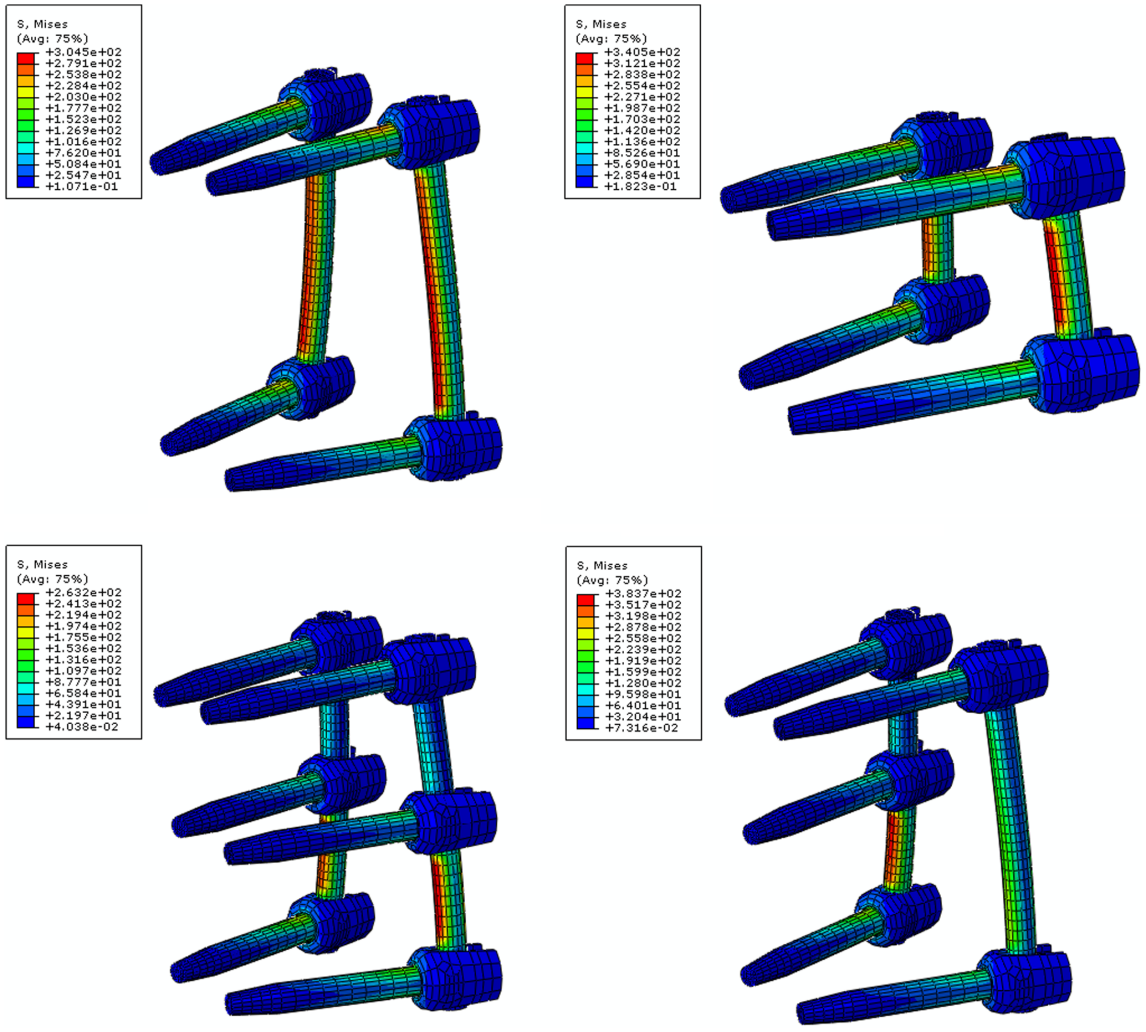
Stress nephogram of the pedicle screws and rods. The stress was concentrated at the screw root under flexion conditions.

The results of the pedicle screw stress distribution are shown in **Fig. 5** **and** demonstrate that the stress was concentrated at the screw root under flexion conditions.

## Discussion

There are several posterior surgical techniques currently employed to treat thoracolumbar burst fractures, and a large number of biomechanical studies suggest that reinforcement with a fracture-level screw combination can help to improve the biomechanical stability [7], [8], [10], [12]. Clinical evidence suggests that reinforcement with a fracture-level screw combination can help to provide better kyphosis correction and that the reinforcement offers immediate spinal stability, more effectively restores fractured vertebral height, and allows earlier ambulation in patients with thoracolumbar burst fracture [7], [9], [11], but no studies have compared the von Mises stresses of the internal fixation devices among different short segment pedicle screw fixation techniques using pedicle fixation at the level of the fracture, especially the mono-segment pedicle screw fixation and intermediate unilateral pedicle screw fixation techniques [13]–[16]. In this study, we investigated the biomechanical comparison of the four posterior short segment pedicle screw fixation techniques used in thoracolumbar burst fractures. The range of motion (ROM), von Mises stress and stress nephogram of the pedicle screws and rods were evaluated.

The finite element model constructed in this study was based on data collected from a spinal CT scan of the patient's thoracolumbar region. The geometric structure of the thoracolumbar vertebrae was generated using medical three-dimensional reconstruction Mimics software. The geometric surface data were preprocessed through smoothing and grid reduction and optimised by Rapidform software to accurately represent the geometry of all models and to minimise the analytic error due to the loss of geometric data. The vertebrae were made up of a solid volume (cancellous bone) and a layer of shell (cortical bone and endplate) with a thickness of 0.4 mm. The intervertebral disc was constructed as a continuum structure that occupied the intervertebral space and was partitioned into annulus fibrosus and nucleus pulposus in approximately 6-to-4 ratio [25]. The annulus fibrosus was modeled as a composite structure: a matrix of annulus ground substance reinforced with inclined fiber trusses which was assigned with a 19% volume of the annulus fibrosus and with an inclination between 15–30 °with respect to the transverse plane [25]. Three-dimensional homogenous and transversely isotropic solid elements were used to model the cortical and cancellous cores. The anterior/posterior longitudinal ligament, intertransverse ligament, ligament flavum, interspinous ligament, and supraspinous ligament were modeled using tension-only truss elements. The constructed T9-L3 finite element model demonstrated accurate simulated geometry and accurate material characteristics and stimulant mechanical characteristics. The inferior endplate of L5 was constrained in all degrees of freedom. A pure moment of 10 Nm combined with a pre-compressive load of 150 N was applied to the top surface of T9. Flexion, extension, left/right lateral bending, and left/right axial rotation were simulated, which was consistent with other experimental models [19], [21]–[23].

In the current study, we attempted to re-create a burst fracture model where the inferior half of T12 and the intervertebral disc between T12 and L1 were completely resected. The construct is highly artificial as depicted, with absence of significant osseous and discal anatomy. We think the spinal fracture model we have built is an extreme case which is similar to A3.1 type that can provide almost little support to the upper vertebrae, and is severe instability, and then we compare the von Mises stresses of the internal fixation devices among different short segment pedicle screw fixation techniques through the extreme spinal fracture model with severe instability. As is known to all, the most common pattern of burst fracture is fracture between the pedicles in the upper half of the body. The spinal fracture model we have built is more severe instability than the most common pattern of burst fracture, we chose the extreme case and carry out our biomechanical study, the conclusions draw from the study on the extreme spinal fracture model with more severe instability can also provide references for clinical treatment of A3.1 type spinal burst fractures.

Compared with the intact normal spine model, the four fixation models showed a decreased range of motion (ROM) in all states of motion. The ROM in flexion, axial rotation, and lateral bending was the smallest in the I6+2 fixation model, followed by the I5+2 and S4+2 fixation models, but the ROM was largest in the M4+2 fixation model. In axial rotation, the ROM could be ranked in the following order from small to large: I6+2, I5+2, S4+2, and M4+2. The results were consistent with previous biomechanical studies that suggested that reinforcement with fracture-level screw combinations can help to improve biomechanical stability [7], [8], [10], [12]. Several clinical studies support the results presented here [7], [9], [11]. Mahar et al. [7] noted that segmental fixation of burst fractures with screws at the level of the fracture offers improved biomechanical stability. Theoretically, segmental fixation provides for additional fixation points that may aid in fracture reduction and kyphosis correction. Guven et al. [9] noted that reinforcement with fracture-level screw combinations can help to provide better kyphosis correction and offers immediate spinal stability in patients with thoracolumbar burst fractures. The findings in Tian's study [11] also indicated that short-segmental fixation combined with intermediate screws more effectively restores fractured vertebral height and allows earlier ambulation compared with conventional intersegmental fixation.

In the current study, the largest maximal von Mises stress of pedicle screws all happened in flexion among all of the fixation models during all states of motion. The value of largest maximal von Mises stress of pedicle screws during all states of motion was largest in the M4+2 fixation model. As shown in the stress distribution results, a maximum level of pedicle screw stress was apparent at the root of the screws under all loading conditions. In clinical practice, the majority of screw breaks occur at this site. These findings were also consistent with the stress features of pedicle screws described by Kim [26]. The largest maximal von Mises stress occurred in the upper pedicle screw in the S4+2 and M4+2 fixation models and in the lower pedicle screw in the I6+2 and I5+2 fixation models. Chen et al. [27] noted that four out of 16 patients who had screws broken had the break occur at the cephalic side. Three out of these four patients experienced an unstable anterior column, as demonstrated by two cases of burst fracture and one case of fracture dislocation. When we suspect that there is pedicle screw broken, we should pay close attention to the root of the upper pedicle screw in the S4+2 and M4+2 fixation techniques and to the lower pedicle screw in the I6+2 and I5+2 fixation techniques.

The value of the largest maximal von Mises stress on rods during all states of motion was the largest in the I5+2 fixation model. The value of the largest maximal von Mises stress of the pedicle screws and rods during nearly all states of motion was smallest in the I6+2 fixation model. The pedicle screws at the fracture site carry some loading stress, thus relieving stress from internal fixation. Therefore, this stress distribution may reduce the possibility of broken or loosened screws. Flexion, extension, right/left lateral bending, and right/left axial rotation tests were conducted to measure the loading forces on the screws. In the current study, we found that additional bilateral pedicle screws at the level of the fracture may result in a stiffer construct and less von Mises stress on pedicle screws compared with traditional short-segment 4 pedicle screw fixation. Index-level pedicle screw fixation to the SPSF decreased the von Mises stress of the pedicle screws; the von Mises stress of the internal fixation was scattered. With pedicle screw fixation at the level of the fracture, the T12 vertebral screw carried a portion of the loading stress. The intermediate screw is thought to function as a push point with an anterior vector, thus creating a lordotic force. The intermediate screw also provides improved “three point fixation”, thus decreasing the cantilever and parallelogram effects and correcting junction kyphosis.

There were a few limitations to this study. The most common pattern of burst fracture is fracture between the pedicles in the upper half of the body. In the current study, we attempted to re-create a burst fracture model where only the inferior half of T12 was resected. In fact the fracture model re-created by the authors was not the most common clinical scenarios. We acknowledge that our model for simulating the fracture is done of for ease of computer simulation, and not a direct reflection of the reality of burst fractures. That's because the fracture model in the current study can be easily created through finite element analysis method and ensure the repeatability of the study, the type of the burst fracture in the current study is similar to A 3.1 (The inferior half of the vertebral body has burst, while the other half remains intact) according to Magerl's classification [28]. The reason is that the inclusion criteria of the patients with spinal fracture who chose the pedicle screw fixation techniques using pedicle fixation at the level of the fracture is the intact of the two pedicles. In the current study, we attempted to re-create a burst fracture model where only the inferior half of T12 and the intervertebral disc between T12 and L1 were completely resected. The most common pattern of burst fracture is fracture between the pedicles in the upper half of the body. The stability of these injuries is reduced in flexion-compression. In particular, fragments of the posterior wall of the vertebral body may be further retropulsed into the spinal canal when the injury is exposed to flexion/compression. The spinal fracture model we created is similar to A3.1 type of fractures (lower half of the vertebral body has burst, while the other half remains intact), but we didn't create sagittal split in the vertebral body, because the purpose of our study is the biomechanical comparisons among different fixation techniques, but not the problem of spinal canal stenosis. It was illustrated by some studies that the pedicle, rather than the vertebral body, contributes approximately 80% of the stiffness and approximately 60% of the pullout strength at the screw-bone interface [29], [30]. So we can see that the integrity of the upper half or inferior half of the body play little effect to the stiffness and pullout strength at the screw-bone interface. We think the spinal fracture model we have built is an extreme case which is similar to A3.1 type that can provide almost little support to the upper vertebrae, and then we compare different short segment pedicle screw fixation techniques through the extreme spinal fracture model with severe instability. We chose the extreme case and carry out our biomechanical study, the conclusions draw from the study on the extreme spinal fracture model with more severe instability can also provide references for clinical treatment of type A 3.1 spinal burst fractures. The other limitations includes absence of statistical analysis and limited sample size (single patient). According to the previous finite element analysis studies [31]–[35], all finite element spine models were reconstructed from single patient's imagine data and the studies were absent of statistical analysis, so we also chose one single healthy patient and there was no statistical analysis in our study, but we think the reviewer's suggestion maybe right if we chose more healthy patients, we can build a more standard healthy spinal model from all the Asian healthy patients' imagine data. The standard Asian healthy spinal finite element model can be used for all the finite element analysis studies in the future. We think it may pay much more manpower, material resources and financial resources to carry out the experiment. In the current study, a finite element model of the T9-L3 spine, which included seven vertebras and six discs, was reconstructed. Geometrical details of T9-L3 spine vertebras were obtained from 64 spiral computed tomography (CT) images of a 40 years old healthy male (65 kg and 175 cm) without a history of spine injury, osteoporosis and radiographic evidence of degenerative sign. The healthy male is a typical case which can represent the mean level of Chinese.

In theoretically, the spine should be a symmetric construct and the ROM and implant stress should be similar in left/right bending or rotation. However, we kept the reference model (normal spine) in its natural configuration of the subject to respect the fact that it was not as symmetry as expected. Despite that, in term of the ROM in the symmetric fixed models (S4+2, M4+2, I6+2), the differences between left and right lateral bending and rotation were less than 5.2% (Fig. 2). In term of the maximal von Mises stress of implants (upper/lower pedicle screws and rods) in the symmetric fixed models (S4+2, M4+2, I6+2), the differences between left and right lateral bending and rotation were less than 15%. Although there was no standard to define the difference in the numerical computation yet, we would like to define that a difference of more than 20% was considered “important” or “relevant”. In the symmetric constructs there were differences between right and left as high as approximately 10% in right/left bending and rotation, but in the asymmetric constructs there were relevant differences between right and left even the largest percent was 41.8% in right/left rotation. For example, compared to the ROM during left lateral bending, the difference between the left and right side were between 1.6%–5.2%. Compared to the von Mises stress of the upper pedicle screw during axial rotation, the difference between the left and right axial rotation were between 0.2%–5.7% in the S4+2, M4+2, I6+2, and 41.8% in the I5+2 fixation models. Compared to the von Mises stress of the rods during lateral bending, the difference between the left and right side were between 0.7%–4.4% in the S4+2, M4+2, I6+2, and 31.9% in the I5+2 fixation models. Comparing the stress between different spinal loadings, the maximal von Mises stress of the implants were observed in flexion in all implanted models. So we can see that there were relevant differences between the left and right side in the asymmetric constructs during some states of motion. Because the finite element spine models were reconstructed from single patient's imagine data. The pedicle dimensions also have an effect on the differences. So the differences are this large in symmetric motion in symmetric constructs in right/left bending and rotation. So it is necessary to investigate several subjects for a more clinically feasible conclusion because the geometry of the vertebrae can affect the analysis result. Possible differences in pedicle dimensions and the forces with and without the posterior ligamentous complex should be regarded as impact factors in the future study. Muscle force and ribs also needs to be considered to simulate more realistic in vivo situations. Possible differences in the screw sizes should be regarded as impact factors in the future study. Finally we want to point out that the spinal fracture model we have built is more severe instability than the most common pattern of burst fracture, we chose the extreme case and carry out our biomechanical study, the conclusions draw from the study on the extreme spinal fracture model can also provide references for clinical treatment of A3.1 type spinal burst fractures, but we can't motivate surgeons to make clinical and surgical decision for a more broad spectrum of burst fractures.

## Conclusions

Additional bilateral pedicle screws at the level of the fracture may result in a stiffer construct and less von Mises stress on pedicle screws and rods compared with SPSF. Index-level pedicle screw fixation to the SPSF decreased the von Mises stress of the pedicle screws, and the von Mises stress of the internal fixation was scattered, thus reducing screw fatigue and breakage. The largest maximal von Mises stress of the pedicle screws during all states of motion were observed in the mono-segment pedicle screw fixation technique. The conclusions draw from the study can provide references for clinical treatment of A3.1 type spinal burst fractures, but we can't motivate surgeons to make clinical and surgical decision for a more broad spectrum of burst fractures.
